# A Comparative Evaluation of Pointing and Crossing in Moving Target Selection

**DOI:** 10.1177/00187208251386219

**Published:** 2025-10-16

**Authors:** Xiaoyu Zhang, Minh Hoang Nguyen, Jin Huang, Huawei Tu

**Affiliations:** 1 2080 La Trobe University, Australia; 2 53036 University of Chinese Academy of Sciences, China

**Keywords:** crossing, pointing, moving target selection, models

## Abstract

**Objective:**

This work presents a comprehensive analysis of fundamental performance of crossing-based moving target selection.

**Background:**

Although the crossing interaction with static targets has been theoretically studied, there has yet to be a generalizable, controlled empirical study investigating the fundamental performance of crossing-based selection for moving targets.

**Method:**

We conducted an experiment with stylus input to investigate how users acquire moving targets with crossing compared to pointing as a baseline.

**Results:**

The most noteworthy finding of our study is that crossing had overall greater advantages over pointing for moving target selection (a 12.37% reduction in task completion time and a 5.88% increase in accuracy rate for *orthogonal crossing*, and a comparable task time and a 4.71% increase in accuracy rate for *collinear crossing*). However, the advantages of crossing would be insignificant when targets become larger than approximately 14.69 mm or move slower than 14.69 mm/s.

**Conclusion:**

Crossing performance varied between *collinear crossing and orthogonal crossing*. $T=a+blog_{2}\left(A+\frac{V}{k}\right)-clog_{2}\left(\frac{W}{2}-\frac{V}{k}\right)$ in (Hoffmann, 1991) can be used to model time performance of crossing-based moving target selection.

**Application:**

Such results provide a theoretical foundation for crossing-based interface design with moving objects.

## Introduction

Moving objects are essential components in many interactive applications such as video games, air traffic control systems, and video surveillance applications. Selecting moving targets in these applications is a mundane but challenging task, as completing such a task requires high sensory-motor coordination of users (Huang et al., 2018, 2019, 2020; Lee et al., 2018; Saunders & Knill, 2003, 2004)—users need to aim at the target visually, estimate its movement trajectory, and acquire it with their motor control ability. The success of acquiring a moving target relies on both accurate spatial and temporal estimation of target position (Huang & Lee, 2019).

A common approach for moving target acquisition is the point-and-click interaction (Huang et al., 2018, 2019). This approach requires the user to move the pointer of an input instrument (e.g., the mouse or the pen) inside the perimeter of the target, and then confirm selection by performing a click action (e.g., pressing the mouse left button or pen tip). To achieve a fast and accurate selection, the visuo-motor system must compensate for the 110 ms reaction delay when planning corrective movements—especially when targets move continuously rather than undergoing discrete displacements as in (Brenner & Smeets, 1997), which forms high demands of both visual attention and motor control. To aid in pointing-based moving target acquisition, many variants of the point-and-click interaction have been proposed by considering the alleviation of visual and motor demands, including increasing the activation area of a moving target by extending a “tail” behind it (Hasan et al., 2011), providing a static proxy of the target to allow for selection without interrupting the target’s motion (Hajri et al., 2011; Hasan et al., 2011), and combining manual pointer-based interaction with eye pointing (Hild et al., 2014).

Crossing has been theoretically investigated with stationary targets (commonly by comparing its performance with pointing) (Accot & Zhai, 2002; Forlines & Balakrishnan, 2008; Luo & Vogel, 2014; Tu et al., 2019; Wobbrock & Gajos, 2008). Results indicate that crossing generally has better or comparable performance to pointing in static target selection. We also anticipate that crossing could outperform pointing in moving target selection. The technique’s proximal positioning with traversal process (landing near the target before stroking through it) could compensate for visuo-motor delays while allowing fine adjustment during traversal—an advantage absent in pointing-based selection. However, there are no generalizable, controlled empirical studies to look into the fundamental performance of selecting moving targets with crossing. We cannot simply justify crossing performance by referring to empirical evidence for crossing interactions with stationary targets for two main reasons. First, the factors involved in the two task types are different. Stationary target selection with crossing mainly considers two target-related factors based on Fitts’ law: target size and distance (Accot & Zhai, 2002; Forlines & Balakrishnan, 2008; Luo & Vogel, 2014; Tu et al., 2019; Wobbrock & Gajos, 2008), while moving target selection usually takes two additional factors into account: target moving speed and direction (Huang et al., 2018, 2019, 2020). The combinational effects of the aforementioned factors may lead to different selection performances relative to crossing-based stationary target acquisition. Second, selecting moving objects is considerably more difficult and error prone than selecting stationary targets, as the former usually needs both spatial and temporal accuracy (Brouwer et al., 2005; Huang & Lee, 2019; Jagacinski et al., 1980) but the latter mainly requires spatial constraint (Fitts, 1954). In summary, without an in-depth understanding of crossing performance in the context of moving target selection, user interface design would not be well grounded with explicit rationales.

Therefore, we conducted an empirical study to investigate crossing performance for moving target selection. Our study was based on the methodology of existing studies on stationary target acquisition with crossing, which generally evaluated crossing performance compared to pointing as a baseline (Accot & Zhai, 2002; Forlines & Balakrishnan, 2008; Luo & Vogel, 2014; Tu et al., 2019; Wobbrock & Gajos, 2008). We aim to address three fundamental research questions. First, how do crossing and pointing fundamentally differ for moving targets? Second, how do target variables (amplitude, width, velocity, movement direction) affect crossing performance? Third, can Fitts’ Law model moving target acquisition via crossing? We carried out an experiment with pen input to look into the performance of crossing vs. pointing in moving target acquisition. We present a thorough report of data analysis and conclude our study with a set of design implications of applying crossing to selection tasks with moving objects.

The contributions of our study are three-fold. First, we conducted a comprehensive fundamental study to explore crossing performance in moving target selection. Second, we analyzed the pros and cons of crossing in the context of moving target acquisition based on experimental data collected. Third, our work contributes new controlled empirical evidence and theoretical results that provide fundamental support for crossing-based moving target selection.

## Related Work

Our study is inspired by works done on moving target acquisition and crossing-based interaction.

### Moving Target Acquisition

Ample research has investigated moving target selection in the field of human movement science (e.g., Brouwer et al., 2005; Mrotek & Soechting, 2007) and the Human–Computer Interaction (HCI) field. Our review focuses on the research in the latter field, which can fall into the following two aspects.

#### Models for Moving Target Selection

Quantitative models have been established to predict time performance of moving target selection with pointing. As a cornerstone of HCI research, Fitts’ law suggests that the time required to point at a target depends on the distance to it (target distance), yet relates inversely to its size (target width) (Fitts, 1954). Fitts’ law has proven to be a robust predictor of pointing time under a wide range of conditions (MacKenzie, 1992). However, it has been insufficient in modeling time of moving target selection (Jagacinski et al., 1980). Therefore, Jagacinski et al. (1980) proposed a model by incorporating target velocity, distance and width, which yielded better goodness of fit than Fitts’ law. Hoffmann (1991) modified Fitts’ Law to fit selection tasks with moving targets by considering system steady state position errors. We evaluated the fitness of three models from (Hoffmann, 1991; Jagacinski et al., 1980) in this study; the results are discussed in Model Fitting Section. Hajri et al. (2011) proposed a new model to quantify the acquisition of 2D moving targets based on Fitts’ Law. However, the model may not be directly applicable to crossing tasks with 1D moving targets without further modification.

Researchers also have focused on modeling the error rate of selecting moving targets in either temporal or spatial constraints. For moving target selection with spatial constraints, Huang et al. (2018) derived a Ternary-Gaussian model to quantify the endpoint distribution of 1D unidirectional moving target selection with pointing, followed by a series of studies to extend the model to 2D moving target selection with pointing (Huang et al., 2019), crossing-based moving target selection (Huang et al., 2020), and targets with different shapes (Zhang et al., 2020). In addition, for moving target selection with temporal constraints, Lee and Oulasvirta (2016) proposed a predictive model for error rates in temporal pointing where the task was to launch a response (e.g., pressing a button) to select a moving target within a limited time frame. Their follow-up study established a cue integration model to account for the effects of both temporal structure cue and visually perceivable movement cue on selection error rates (Lee et al., 2018). By combining the Ternary-Gaussian model (Huang et al., 2018) and the cue integration model (Lee & Oulasvirta, 2016), Huang and Lee (2019) proposed a model to predict pointing error rates for the selection of moving targets in both spatial and temporal dimensions simultaneously. Yamanaka et al. (2020) introduced a Servo-Gaussian model to predict success rates in continuous manual tracking tasks. Lee et al. (2019) established a predictive model of latency on error rates with a method to compensate for latency effects in moving target selection games. Do et al. (2021) proposed a simulation model for point-and-click operations with stationary or moving objects, which could predict trial completion time, distribution of click endpoints and cursor trajectories.

Predicting intended moving targets is also of high interest to researchers. Casallas et al. (2013) proposed an approach to estimate user’s intention to select a moving target by using decision trees with features based on target distance and size. Ortega (2013) determined the target of interest with target-distance-based scores computed by a heuristic method. Based on the above two approaches, Casallas et al. (2014) integrated head-pose-related features with both target size and distance to predict intended moving targets.

#### Techniques for Moving Target Selection

Moving target selection techniques can be designed based on interaction paradigms such as pointing, tracing and crossing.

Pointing is a fundamental paradigm and users can benefit from being able to directly select one or more moving targets. With such an approach, users need to continually track the target and simultaneously plan to select it by moving an input device (e.g., the pen or mouse) over its perimeter. However, this approach usually suffers from low accuracy and long times (Hasan et al., 2011), especially for small or fast-moving targets (Hajri et al., 2011). Therefore, many variants of direct pointing have been proposed to assist in moving target selection, such as creating static proxies of moving objects in the scene (Hasan et al., 2011), enlarging moving objects’ activation area (Grossman & Balakrishnan, 2005; Hasan et al., 2011), temporarily pausing moving objects’ motion (Hajri et al., 2011; Ragan et al., 2020), combining gaze input for pointer positioning (Hild et al., 2014, 2016), or model-driven techniques based on selection endpoint distributions (Huang et al., 2018; Li et al., 2018).

Tracing along the trajectory of a moving target can be an alternative to pointing for moving target selection (e.g., Khamis et al., 2018). Such a method is also called *motion correlation* (Velloso et al., 2017). Objects are represented by motion in the interface and the user identifies a target by mimicking its specific motion; selection thus relies on the match between the motions of the target and the user. A detailed review can be found in (Velloso et al., 2017).

Crossing is also a promising paradigm for target selection. Prior studies on crossing-based target selection are mainly focused on tasks with stationary targets (e.g., Accot & Zhai, 2002; Forlines & Balakrishnan, 2008; Luo & Vogel, 2014; Tu et al., 2019; Wobbrock & Gajos, 2008). A noteworthy exception is the study by You et al. (2014), which proposed a technique to select a moving target by crossing an expanding wave pattern attached to the target on augmented reality devices.

### Crossing-Based Interaction

An early introduction of the crossing paradigm was initiated in Accot and Zhai’s study of steering tasks (Accot & Zhai, 1997), in which they devised the steering law (it predicts cursor movement time through constrained paths (tunnels) based on path length and width) based on a goal-crossing experiment. To explore user performance of crossing-based interaction, they systematically investigated four types of crossing tasks in comparison to two pointing tasks (Accot & Zhai, 2002). Since then, crossing has gained considerable attention from the viewpoints of fundamental performance evaluation and interaction technique design, which are reviewed respectively below.

Researchers have conducted fundamental performance evaluation for mouse input (Wobbrock & Gajos, 2008), stylus interaction (Accot & Zhai, 2002; Apitz & Guimbretière, 2004; Forlines & Balakrishnan, 2008; Huang et al., 2020; Tu et al., 2021; Yamanaka & Stuerzlinger, 2020), direct finger touch (Luo & Vogel, 2014), remote display interaction (Nakamura et al., 2008), virtual reality (Huang et al., 2019; Tu et al., 2019; Yan et al., 2020), and augmented reality (Uzor & Kristensson, 2021). Generally, crossing had comparable, if not superior, performance than pointing, thus can substitute or complement pointing for interaction technique design. Note that these studies were focused on stationary targets and their results may not be generalized to moving target acquisition. To the best of our knowledge, the only fundamental study was conducted by Huang et al. (2020), which proposed a Quaternary-Gaussian model to quantitatively measure the endpoint uncertainty of moving target selection with crossing. However, their study did not look into user performance of crossing (e.g., task time) with a comparative evaluation between crossing and pointing.

Crossing has gained wide adoption in interaction technique design. Crossing can be used in conjunction with other paradigms such as pointing (Luo & Vogel, 2015; Sidenmark et al., 2021), dragging (Dragicevic, 2004), and gesturing (Choe et al., 2009) to offer new interaction styles. An important application lies in target selection, either with discrete crossing (Apitz & Guimbretière, 2004) or continuous crossing (Apitz & Guimbretière, 2004; Dixon et al., 2008; Perin & Dragicevic, 2014). Especially, crossing has been applied to moving target selection as an alternative to pointing (Fruit Ninja, 2021; You et al., 2014).

Our review shows that moving target selection has been an important task in HCI and crossing has served as a viable method for such a task. However, there is no study systemically exploring fundamental performance on crossing-based moving target selection. Our study thus aims to provide a generalizable and empirical investigation to fill this gap.

## Experiment

The experiment was designed to compare the performance of pointing and crossing when selecting moving targets in specified directions. Such a design has been adopted in previous studies (Huang et al., 2018, 2020) and is also a representative user interface such as in traffic monitoring systems with oriented lanes (You et al., 2014).

### Apparatus

The experiment was performed on a Wacom DTH-W1310P Tablet PC, which ran Microsoft Windows 8.1 on an Intel i7-5557U CPU and 8 GB memory and had a 13.3-inch display having a screen resolution of 2560 × 1440 pixels with a 60 Hz refresh rate. It offered both stylus and touch input. In the experiment, the stylus was used in the direct input mode to perform both pointing and crossing tasks. It is a common device for pointing tasks (Ren & Moriya, 2000) and is also naturally suitable for drawing strokes (Tu et al., 2015). The experiment program was developed in C#.

### Participants

We recruited 15 right-handed participants (5 female) with a mean (±SD) age of 23.75 ± 0.83 years from the local campus for the experiment. Only one had experience in using pen-based devices such as tablet PCs. This research complied with the tenets of the Declaration of Helsinki and was approved by the Institutional Review Board at University of Chinese Academy of Sciences. Informed consent was obtained from each participant.

### Experiment Design

The experiment was a within-subject repeated measures design with the independent and dependent variables below.

#### Independent Variables

**Figure 1.**
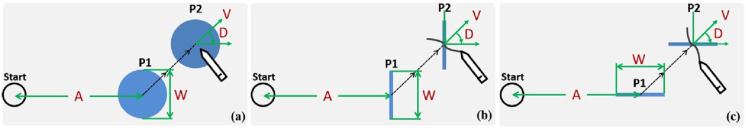
Illustration of task interfaces in the experiment. (a) Pointing: targets are blue circles. (b) Orthogonal crossing and (c) collinear crossing: targets are blue bars. P1 indicates the initial position of the target (target center). P2 indicates target position (target center) when it is selected. Tapping the “Start” circle is to start a trial. A, W, D, and V represent initial target amplitude, target width, movement direction, and velocity, respectively. Note that besides the line under the pen tip in (b) and (c), all characters and lines were not shown on the experiment interface.

The independent variables are initial target amplitude (*A*), target width (*W*), movement direction (*D*), movement velocity (*V*), and task type (Figure 1). The values for each variable were selected based on Huang et al.’s (2020) study with modifications to fit the requirements of our study. We used mm, an absolute unit of length, to ensure cross-device consistency in our experiment design.

Initial target amplitude (*A*) was the distance between the center of the “Start” circle and the initial target position (target center: P1 in Figure 1). It had two levels: 88.13 mm and 176.26 mm.

Target width (*W*) was the length of the bar for crossing tasks and the diameter of the circle for pointing tasks. It had three levels: 4.90 mm, 7.34 mm, and 14.69 mm.

Target movement direction (*D*) was the direction of the ray from the target center in P1 to the target center in P2 (Figure 1). It had eight levels, ranging from E (0°) to ESE (315°) in 45° increments. *D* was constant in each single trial.

Target movement velocity (*V*) was the magnitude of the target’s velocity. It had three levels: 14.69 mm/s, 29.38 mm/s, and 58.75 mm/s. Modeling the Endpoint Uncertainty in Crossing-Based Moving Target Selection was constant in each single trial.

Task type included pointing, orthogonal crossing, and collinear crossing. The pointing task was a variant of task design in (Huang et al., 2019) with target movement explicitly specified in eight directions in our experiment (Figure 1(a)). The pen tip should click inside the target for selection. *Orthogonal crossing* and *collinear crossing* are representative tasks used in previous crossing studies (Accot & Zhai, 2002; Forlines & Balakrishnan, 2008; Luo & Vogel, 2014; Tu et al., 2019). *Orthogonal crossing* derives its name from the initial perpendicular orientation of the target relative to the “Start” circle-target center reference line (Figure 1(b)). Selection requires a rightward crossing stroke. *Collinear crossing* features targets initially aligned collinearly with this reference line (Figure 1(c)), and requires a downward crossing stroke for selection. We investigated *orthogonal* and *collinear crossing* for two primary reasons. First, these techniques rely on distinct motor behaviors during target acquisition. Crossing-based selection comprises two phases: coarse positioning (moving near the target) and fine positioning (traversing its boundary). *Orthogonal crossing* typically preserves movement momentum across the two phases, whereas *collinear crossing* requires a pronounced directional shift in the motion trajectory between phases. Second, both techniques serve as representative tasks in foundational crossing research (Accot & Zhai, 2002; Forlines & Balakrishnan, 2008; Luo & Vogel, 2014; Tu et al., 2019).

Compared to previous pointing vs. crossing studies, our study had two special considerations. First, the target shapes selected for comparing pointing and crossing interactions reflect each technique’s inherent characteristics. As established in pointing literature (MacKenzie, 1992), target shape complicates effective width determination across movement directions. Circular targets mitigate this challenge by maintaining constant width (diameter) regardless of approach angle, hence have been used in previous studies on moving target selection (Tu et al., 2019; Uzor & Kristensson, 2021; Wobbrock & Gajos, 2008). However, they are not “interaction friendly” for crossing tasks due to requiring full traversal of the circular area. Following previous crossing research (Accot & Zhai, 2002; Huang et al., 2020; Tu et al., 2019; You et al., 2014), we employed bar targets, which aligns with crossing’s core interaction principle: target acquisition via boundary intersection. Crucially, both circular and bar targets share a single control parameter (width), enabling methodologically equitable comparisons between techniques based on Fitts’ law. Second, as in previous studies of moving target selection (Huang et al., 2018, 2020), the start position was to the left of the target. So the macro movement of the stylus was from left to right, which is in line with the common left-to-right writing direction. We did not include other target positions (e.g., the start position was to the right of the target) as this could complicate our experiment design given that the experiment already had five independent variables. Future work will examine the effects of target position on pointing vs. crossing in moving target selection.

#### Dependent Variables

The dependent variables are task completion time, selection accuracy rate and subjective feedback. Task completion time for a trial was defined as the duration from clicking the “Start” circle to pointing at or crossing a moving target. For pointing, a trial ends when lifting the pen tip from the screen after clicking the target. For crossing, a trial finishes when the pen tip traverses the target boundary. A pointing was successful if the pointing position was inside the target’s area. A crossing was successful if a line segment formed between any two successive touch points intersected with the line segment representing the target. Selection accuracy rate was calculated as the ratio of the number of successful trials to the number of total trials. The unsuccessful trials were excluded in time analysis as previous studies (Accot & Zhai, 2002; Luo & Vogel, 2014; Tu et al., 2019).

### Task and Procedure

A prestudy questionnaire was administered to collect participants’ demographic data and experience in using pen-based devices. Participants sat in a chair and performed the experiment task with the stylus offered by the tablet PC placed on a desk. The experiment consisted of a practice session and a test session. In the practice session, participants were first introduced to the task. They needed to click the “Start” circle to begin a trial (along with the circle disappearing), and then point at or cross a moving target as accurately and quickly as possible (Figure 1). The target turned from blue to green once successfully selected. In case of an error, an error tone sounded to remind participants to improve accuracy and participants continued the trial until the pen tip passed or hit the target.

In the formal test, participants were required to perform the three task types in an order counterbalanced across participants using a Latin square design. Each task type had 3 blocks of 144 sets of combinations of target amplitude, target width, movement direction, and movement velocity. For each set of combinations, participants were required to perform 2 trials of target selection (measured data trials) following the same instructions in the practice session. The order of trials in a block was randomized. Participants took a 1-min break between blocks, and a 1-min break between task types. In summary, the experiment had (excluding practice trials) 15 participants × 3 task types × 144 condition combinations (2 target amplitudes × 3 target widths × 8 movement directions × 3 movement velocities) × 2 repeats of measured data trials × 3 blocks = 38,880 trials (2592 trial per participant).

After completing the experiment task, each participant was asked to fill in a questionnaire to rate the three task types on 7-point Likert Scales regarding selection accuracy, selection speed, and ease of use (7 for highest preference and 1 for lowest preference). The participants took approximately 80 min (including breaks) to finish the experiment.

## Data Analysis

For the 3 task types × 144 condition combinations, we first removed the outliers (1.26% of the data) with more than three standard deviations from mean time. For both task completion time and selection accuracy rate, the data passed the Kolmogorov–Smirnov test (α = 0.05) for normality of the distribution. We thus analyzed both task completion time and selection accuracy rate using repeated measures ANOVA and post hoc comparisons with Bonferroni adjustment.

We then checked the learning effects over the three blocks for each task type in terms of task completion time. For all the three tasks, the block did not have significant main effects on completion time (all *p* > 0.05). Participants had already reached a steady performance after practice. Hence, we used the data of the three blocks for data analysis.

### Task Completion Time

We calculated the main effects of the five independent variables on task completion time. Task type had a significant main effect on task time ( $F_{2,28}=13.03,p\;<\;0.01,\eta_{p}^{2}=0.48$ ). Post hoc tests revealed that *orthogonal crossing* (M = 722 ms, SD = 96 ms) had significantly shorter times than both *collinear crossing* (M = 803 ms, SD = 105 ms) and *pointing* (M = 823 ms, SD = 91 ms) (both *p* < 0.01), and the latter two tasks did not significantly differ in task time (*p* = 1.00). Initial target amplitude had a significant main effect on task time ( $F_{1,14}=232.19,p\;<\;0.01,\eta_{p}^{2}=0.94$ ). As expected, the small amplitude (M = 704 ms, SD = 73 ms) resulted in shorter times than the large one (M = 862 ms, SD = 101 ms). Target width had a significant main effect on task time ( $F_{2,28}=94.21,p\;<\;0.01,\eta_{p}^{2}=0.81$ ). The small width led to the longest time (M = 890 ms, SD = 125 ms), followed by the medium width (M = 801 ms, SD = 89 ms), and the large width (M = 683 ms, SD = 52 ms) (all *p* < 0.01). Target movement velocity had a significant main effect on task time ( $F_{2,28}=3.56,p\;<\;0.05,\eta_{p}^{2}=0.20$ ). However, there were no significant differences between any two of the three target velocities (low: M = 786 ms, SD = 81 ms; medium: M = 786 ms, SD = 85 ms; high: M = 773 ms, SD = 91 ms) (all *p* > 0.08). Target movement direction had a significant main effect on task time ( $F_{7,98}=9.23,p\;<\;0.01,\eta_{p}^{2}=0.40$ ). Significant differences were observed between 0° and 180°, 0° and 225°, 0° and 270°, 45° and 270°, and 270° and 315° (all *p* < 0.05).

**Figure 2.**
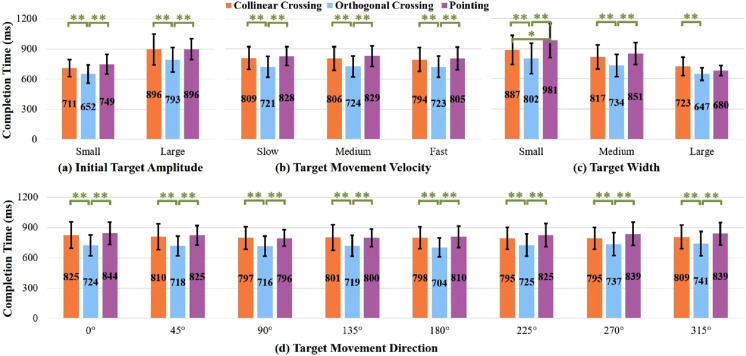
Task completion time for each task type in different (a) initial target amplitudes, (b) target velocities, (c) target widths, and (d) target movement directions. Numbers in the bar center are mean values (ms). * and ** represent *p* < 0.05 and *p* < 0.01, respectively. Error bars represent 0.95 confidence interval.

There were significant interaction effects between task type and initial target amplitude ( $F_{2,28}=5.41,p\;<\;0.05,\eta_{p}^{2}=0.28$ ), between task type and target movement velocity ( $F_{4,56}=5.17,p\;<\;0.01,\eta_{p}^{2}=0.27$ ), between task type and target width ( $F_{4,56}=35.05,p\;<\;0.01,\eta_{p}^{2}=0.72$ ), and between task type and target movement direction ( $F_{14,196}=5.10,p\;<\;0.01,\eta_{p}^{2}=0.27$ ). Due to these interaction effects, we further analyzed the effects of task type on task time on a per target amplitude basis, a per movement velocity basis, a per target width basis and a per target movement direction basis respectively (Figure 2).

For both small and large initial target amplitudes, task type had a significant main effect on task time (small: $F_{2,28}=13.96,p\;<\;0.01,\eta_{p}^{2}=0.50$ ; large: $F_{2,28}=11.24,p\;<\;0.01,\eta_{p}^{2}=0.45$ ). Generally, *collinear crossing* and *pointing* had similar task times (all *p* > 0.24), and both had significantly longer times than *orthogonal crossing* (all *p* < 0.01).

For the three levels of target movement velocity, task type had a significant main effect on task time (slow: $F_{2,28}=14.21,p\;<\;0.01,\eta_{p}^{2}=0.50$ ; medium: $F_{2,28}=13.00,p\;<\;0.01,\eta_{p}^{2}=0.48$ ; fast: ${\;F}_{2,28}=10.04,p\;<\;0.01,\eta_{p}^{2}=0.42$ ). Overall, *orthogonal crossing* had significantly shorter times than both *collinear crossing* and *pointing* (all *p* < 0.01), while the latter two tasks did not significantly differ in task time (all *p* > 0.65).

For the three levels of target width, task type had a significant main effect on task time (small: $F_{2,28}=21.37,p\;<\;0.01,\eta_{p}^{2}=0.60$ ; medium: $F_{2,28}=15.47,p\;<\;0.01,\eta_{p}^{2}=0.53$ ; large: $F_{2,28}=10.39,p\;<\;0.01,\eta_{p}^{2}=0.43$ ). In general, *orthogonal crossing* was significantly faster than *collinear crossing* (all *p* < 0.01). The differences between *orthogonal crossing* and *pointing* varied depending on target width. For both small and medium target widths, *orthogonal crossing* had significantly shorter times than *pointing* (both *p* < 0.01), but similar times for the large target width (*p* = 0.13). In addition, the differences between *collinear crossing* and *pointing* changed for different target widths. *Collinear crossing* had significantly shorter times than *pointing* for the small target width (*p* < 0.05), but comparable time for both medium and large target widths (*p* > 0.14).

For the eight levels of target movement direction, task type had a significant main effect on task time (all *p* < 0.01). Generally speaking, *orthogonal crossing* had significantly shorter times than both *collinear crossing* and *pointing* (all *p* < 0.01), and the latter two tasks did not significantly differ in task time (all *p* > 0.32).

### Selection Accuracy Rate

We analyzed the main effects of the five independent variables on selection accuracy. There was a significant main effect for task type on selection accuracy rate ( $F_{2,28}=32.43,p\;<\;0.01,\eta_{p}^{2}=0.70$ ). Post hoc tests revealed that *pointing* (M = 0.85, SD = 0.04) had significantly lower selection accuracy than both *collinear crossing* (M = 0.89, SD = 0.03) and *orthogonal crossing* (M = 0.90, SD = 0.03) (both *p* < 0.01). However, the two crossing types did not differ in selection accuracy (*p* = 0.94). Initial target amplitude did not have a significant main effect on selection accuracy rate ( $F_{1,14}=0.73,p=0.41,\eta_{p}^{2}=0.05$ ). The mean accuracy (SD) was 0.88 (0.03) for the small amplitude and 0.88 (0.03) for the large amplitude, respectively. Target movement velocity had a significant main effect on selection accuracy rate ( $F_{2,28}=324.77,p\;<\;0.01,\eta_{p}^{2}=0.96$ ). As expected, the low movement velocity led to the highest accuracy (M = 0.95, SD = 0.03), followed by the medium velocity (M = 0.92, SD = 0.03), and then the high movement velocity (M = 0.77, SD = 0.05) (all *p* < 0.01). Target width had a significant main effect on selection accuracy rate ( $F_{2,28}=207.99,p\;<\;0.01,\eta_{p}^{2}=0.94$ ). Selection accuracy increased from the small target width (M = 0.77, SD = 0.06), the medium target width (M = 0.89, SD = 0.03), to the large target width (M = 0.98, SD = 0.01) (all *p* < 0.01). Target movement direction had a significant main effect on selection accuracy rate ( $F_{7,98}=2.44,p\;<\;0.05,\eta_{p}^{2}=0.15$ ). Post hoc tests showed no significant differences between each two levels of movement direction (all *p* > 0.18).

**Figure 3.**
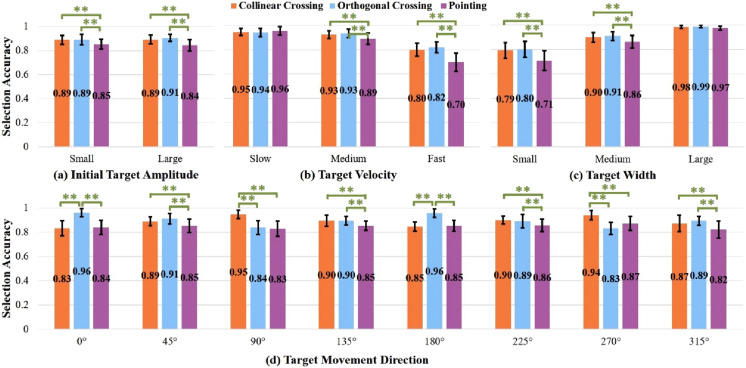
Selection accuracy rate for each task type in different (a) initial target amplitudes, (b) target velocities, (c) target widths and (d) target movement directions. Numbers in the bar center are mean values. ** represents *p* < 0.01. Error bars represent 0.95 confidence interval.

There was no significant interaction effect between task type and initial target amplitude ( $F_{2,28}=2.46,p=0.10,\eta_{p}^{2}=0.15$ ). However, there was a significant interaction effect between task type and target movement velocity ( $F_{4,56}=43.11,p\;<\;0.01,\eta_{p}^{2}=0.76$ ), between task type and target width ( $F_{4,56}=16.21,p\;<\;0.01,\eta_{p}^{2}=0.54$ ), and between task type and target movement direction ( $F_{14,196}=33.51,p\;<\;0.01,\eta_{p}^{2}=0.71$ ). Therefore, we further analyzed the effects of task type on selection accuracy rate for each initial target amplitude, target movement velocity, target width, and target movement direction, respectively (Figure 3).

For both small and large initial target amplitudes, task type had a significant main effect on selection accuracy rate (small: $F_{2,28}=11.50,p\;<\;0.01,\eta_{p}^{2}=0.45$ ; large: $F_{2,28}=32.74,p\;<\;0.01,\eta_{p}^{2}=0.70$ ). *Pointing* had significantly lower selection accuracy than both *collinear crossing* and *orthogonal crossing* (all *p* < 0.01). However, the two crossing types did not differ in selection accuracy (all *p* > 0.26).

The effects of task type on selection accuracy varied depending on target movement velocity. For the low target movement velocity, task type did not have a significant main effect on selection accuracy rate ( $F_{2,28}=2.26,p=0.12,\eta_{p}^{2}=0.14$ ). Post hoc tests revealed that there were no significant differences between each two of the three task types (all *p* > 0.24). For both medium and high target movement velocities, task type had a significant main effect on selection accuracy rate (medium: $F_{2,28}=15.90,p\;<\;0.01,\eta_{p}^{2}=0.53$ ; high: $F_{2,28}=60.34,p\;<\;0.01,\eta_{p}^{2}=0.81$ ). *Orthogonal crossing* had similar selection accuracy as *collinear crossing* (*p* > 0.28), and both had significantly higher selection accuracy than pointing (both *p* < 0.01).

The effects of target type on selection accuracy remained consistent across the small and medium target widths. Task type had significant main effects on selection accuracy rate (small: $F_{2,28}=35.59,p\;<\;0.01,\eta_{p}^{2}=0.72$ ; medium: ${\;F}_{2,28}=10.69,p\;<\;0.01,\eta_{p}^{2}=0.43$ ). The selection accuracy of *collinear crossing* was similar to *orthogonal crossing* (all *p* > 0.60), and both crossing types led to significantly higher accuracy than pointing (all *p* < 0.01). For large target width, while task type had a significant main effect on selection accuracy rate as well ( $F_{2,288}=9.31,p\;<\;0.01,\eta_{p}^{2}=0.40$ ), the three tasks did not differ in accuracy (all *p* < 0.23).

Generally, for the eight levels of target movement direction, task type had a significant main effect on selection accuracy rate (all *p* < 0.05). However, the effects of task type on selection accuracy varied depending on target movement direction. When targets moved along the horizontal direction (either right (0°) or left (180°)), *orthogonal crossing* had significantly higher accuracy than both *collinear crossing* and *pointing* (all *p* < 0.01), and the latter two tasks did not significantly differ in selection accuracy (all *p* = 1.00). When targets moved diagonally (i.e., 45°, 135°, 225°, and 315°), *orthogonal crossing* had similar selection accuracy as *collinear crossing* (all *p* > 0.26), and both crossing types had higher accuracy than *pointing* (all *p* < 0.01). When targets moved along the vertical direction (either downward (90°) or upward (270°)), *collinear crossing* was significantly more accurate than both *orthogonal crossing* and *pointing* (all *p* < 0.01), while the latter two tasks had similar accuracy (both *p* > 0.42).

### Model Fitting

As stated in Models for Moving Target Selection Section, we used the data from this experiment to evaluate the fitness of the three formulae for selection time (Hoffmann, 1991; Jagacinski et al., 1980):

(1) $$T=a+\text{bA}+c\left(V+1\right)\left(\frac{1}{W}-1\right)$$

(2) $$T=\frac{1}{k}\ln\left(\frac{A+\frac{V}{k}}{\frac{W}{2}-\frac{V}{k}}\right)$$

(3) $$T=a+blog_{2}\left(A+\frac{V}{k}\right)-clog_{2}\left(\frac{W}{2}-\frac{V}{k}\right)$$

In all formulae, T is target selection time, V is target velocity, A is the initial amplitude (at time = 0), W is target width, and a , b , c , and k are empirically determined constants.

Table 1 shows the parameter estimates and standard errors for those estimates, $R^{2}$ values and AIC values for the regression. Overall, equation (3) had higher fitness values than both equations (1) and (2) across the three task types, indicating the advantage of equation (3). Equation (2) yielded better goodness of fit than equation (1) for both *pointing* and *orthogonal crossing*, but worse goodness of fit for *collinear crossing*. In addition, we also calculated Akaike information criterion (AIC) values (Akaike, 2003) to check the risk of overfitting of the models. As illustrated in Table 1, equation (3) generally had smaller AIC values than the other two models. Based on the small-is-better rule of AIC analysis, the results further verify the advantage of equation (3) in time prediction of moving target selection.

**Table 1.** Summary of Model Fitting Results.

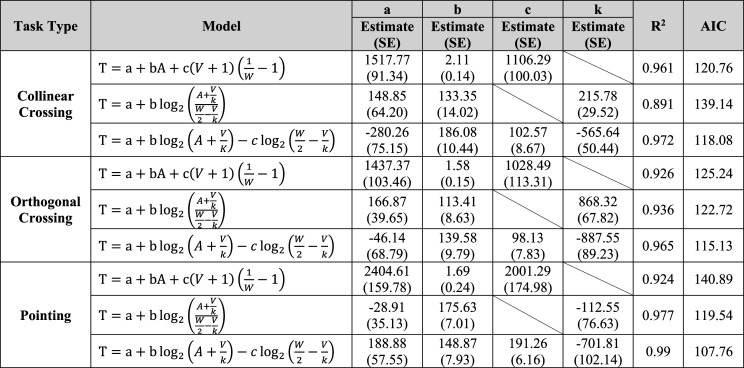

Due to the advantage of equation (3), we further look into the breakdown of equation (3). According to Hoffmann’s explanation (Hoffmann, 1991), equation (3) has two components: the initial distance-covering phase ( $\log_{2}\left(A+\frac{V}{k}\right)$ ) and the accuracy phase ( $\log_{2}\left(\frac{W}{2}-\frac{V}{k}\right)$ ) (Hoffmann, 1991). The first phase refers to approaching the target from the starting position and the second phase refers to finalizing target selection. The coefficient b and c reflect the information processing rate of the first and second phases, respectively; the larger the value, the lower rate of processing information on the phase. In Table 1, *orthogonal crossing* has the smallest value of both b and c , indicating the highest information processing rate on both phases when compared with the other two task types. *Collinear crossing* has a higher value of *b* but a smaller value of *c* than *pointing*, which means that *collinear crossing* resulted in a lower information processing rate on the initial distance-covering phase of the movement but a higher information processing rate on the accuracy phase. Overall, both *crossing* types led to higher rates of processing information on the accuracy phase of the movement.

### Subjective Preference

**Figure 4.**
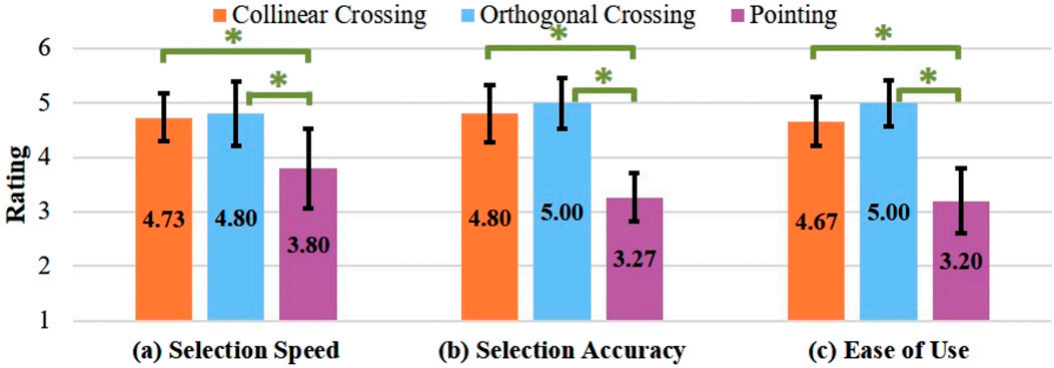
Subjective preference for each crossing type in terms of (a) selection speed, (b) selection accuracy, and (c) ease of use. Numbers in the bar center are mean values. * represents *p* < 0.05. Error bars represent 0.95 confidence interval.

As the data of subjective preference were not normally distributed, we applied the Friedman test with post hoc analysis (Wilcoxon signed-rank test) to analyze the data. There was a significant difference between the task types in selection speed ( $\chi^{2}$ = 6.38, p < 0.05$), selection accuracy ( $\chi^{2}$ = 16.96, p < 0.01), and ease of use ( $\chi^{2}$ = 17.29, p < 0.01). As illustrated in Figure 4, for the three measures, post hoc comparisons showed that both crossing types were rated significantly higher than pointing (all p < 0.05), but there was no significant difference between *collinear crossing* and *orthogonal crossing* (all p > 0.21). Generally speaking, participants favored crossing over pointing. The two crossing types were regarded as equivalent to each other in terms of selection performance, even though they differed in task completion time according to the experimental data analysis.

## Discussion

This study investigated fundamental performance of *crossing* in moving target selection by comparing to *pointing* as a baseline. Generally, *crossing* showed advantages over *pointing* in selection efficacy and was preferred by the participants. Result analysis revealed that *orthogonal crossing* had overall shorter selection times than *pointing*. Such a time advantage of *orthogonal crossing* can be supported by the coefficient analysis of equation (3), which showed that *orthogonal crossing* had higher rates of processing information on both initial distance-covering and landing-crossing phases than *pointing*. *Collinear crossing* had similar task time when compared to *pointing*. According to the coefficient analysis of equation (3), *collinear crossing* had lower rates of processing information on the initial distance-covering phase but higher rates on the landing-crossing phase. This may account for the comparative time performance between *collinear crossing* and *pointing*.

Both *crossing* types had higher accuracy relative to *pointing*. This is in line with the coefficient analysis of equation (3), which revealed that both *crossing* types had higher processing information rates on the accuracy phase than *pointing*. A previous study also showed that *crossing* could reduce error rates of moving target selection substantially as against pointing-based techniques (≥61.75%) (You et al., 2014). Our data analysis shows that the duration of the landing-stroking process (i.e., landing the pen in proximity to the target and then stroking through it) accounted for 11.03% (89 out of 803 ms) and 14.55% (105 out of 722 ms) of the total task time for *collinear crossing* and *orthogonal crossing* respectively. Such a duration may compensate for the visuo-motor delay for moving target selection (approximately 110 ms as specified in (Brenner & Smeets, 1997)) and leave some leeway for the fine adjustment of the stroking operation, which may in turn improve selection accuracy. In summary, the above analysis of both selection time and accuracy indicates that *crossing* is a practical substitute for *pointing* for moving target acquisition.

While the differences between *pointing* and *crossing* did not significantly vary across most target-related factors, there are some exceptional cases which are worthy of discussion. *Orthogonal crossing* generally outperformed *pointing* in moving target selection, but they had comparable performance in two conditions: similar task time for the large target width (14.69 mm) as well as similar selection accuracy for both large target width (14.69 mm) and low target movement velocity (14.69 mm/s). In addition, *collinear crossing* had overall similar task time and higher selection accuracy than *pointing*, but their accuracy rates did not differ in the large target width (14.69 mm) and the low target movement velocity (14.69 mm/s). Seemingly, *crossing* has general advantages over *pointing*, but such advantages are not marked for the large target width and low target movement velocity.

We compared the performance between *orthogonal crossing* and *collinear crossing* to gain insights into the two crossing types for moving target selection. Generally speaking, *orthogonal crossing* resulted in shorter task times and similar selection accuracy than *collinear crossing*. When the target was orthogonal to the line connecting the start position and the target center in the initial position (*orthogonal crossing*), users could plan and execute crossing with less effort than *collinear crossing*. This can be underpinned by the coefficient analysis of equation (3), which revealed that *orthogonal crossing* had generally higher rates of processing information than *collinear crossing*. We analyze differences between *orthogonal crossing* and *collinear crossing* by deconstructing target selection into two phases: coarse positioning (moving the pointer near the target) and fine positioning (traversing the target boundary). We argue that orthogonal crossing enables smoother phase transitions than collinear crossing. From the viewpoint of motor control, *orthogonal crossing* primarily employs continuous elbow rotation, sustaining movement momentum. Conversely, *collinear crossing* demands more complex coordination: coarse positioning mainly relies on elbow rotation, while fine positioning requires combined elbow-wrist action for downward traversal. These motor control differences likely underlie performance variations between techniques. Interestingly, such results are contrary to the conclusions of *orthogonal crossing* vs. *collinear crossing* in stationary target selection. According to the pen-based crossing study (Apitz et al., 2008), *orthogonal crossing* had significantly longer times than *collinear crossing*. The perceptual motor mechanism of crossing-based interaction may be influenced by target motion properties (i.e., moving or stationary).

## Implications for Design

Based on the data analysis, we provide the following general design principles and practical design examples for crossing-based moving target selection. Note that the principles are applicable to pen-based crossing interaction with target bars as this study was conducted with that interface. To apply the principles to targets with other shapes or other input devices, we need to conduct further comparative studies between *crossing* and *pointing* with those target shapes and input devices based on our methodology.

### General Design Principles

First, both types of *crossing* generally outperformed *pointing* for moving target selection. Therefore, we suggest using *crossing* in applications requiring the selection of moving targets. Especially, *crossing* can significantly improve the accuracy of moving target acquisition over *pointing* and should be considered as the primary design option for user interfaces demanding high accuracy of target selection.

Second, *orthogonal crossing* resulted in overall shorter task times and similar selection accuracy than *collinear crossing*. For selecting moving targets with a specified route (e.g., in traffic video monitoring applications), if the position of the target-selection pointer needs to be set (e.g., homing after completing a task), it should be placed perpendicular to the route, rather than parallel to.

Third, our results support $T=a+blog_{2}\left(A+\frac{V}{k}\right)-clog_{2}\left(\frac{W}{2}-\frac{V}{k}\right)$ in Hoffmann (1991) over competing models, so we endorse its use in interface design and analysis.

Fourth, when targets become larger than approximately 14.69 mm or move slower than 14.69 mm/s, *crossing* does not show significant advantages over *pointing*. Thus, the two techniques could be equally treated for moving target selection in terms of selection efficacy.

### Practical Design Examples

#### Object Tail

**Figure 5.**
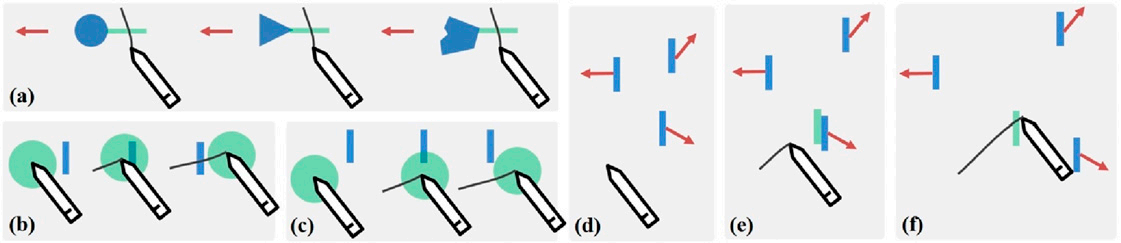
Illustration of design examples. Object tail (a): tails can be attached to objects with different shapes. Fat pointer: the pointer (b) fully or (c) partly covers the object for selection. Object hook: (d) the pen initiates a stroke; (e) the closest object ahead of the pointer along its movement direction is engaged as a target of interest and a proxy is generated; (f) crossing the proxy to select the target. Note that arrow lines represent target movement directions.

Inspired by the “comet” technique for pointing-based moving target selection (Hasan et al., 2011), we can enhance a moving object by attaching a bar-like tail to it (Figure 5(a)), so as to enlarge its activation area and also make it more “friendly” for crossing. As our results indicated that larger target width tended to have shorter selection times and higher selection accuracy, we would expect the object-tail design could improve moving target acquisition with crossing.

#### Fat Pointer

This technique is based on the area cursor (Worden et al., 1997), which has a large hotspot defined by the boundary of the pointer. An object is selected when the “fat pointer” moves from one side of the object to the other with the “fat pointer” fully containing (Figure 5(b)) or partially intersecting with the object (Figure 5(c)). This is like enlarging the object’s activation size, hence may lead to faster selection with higher accuracy. Further design considerations can dynamically resize the area of the “fat pointer” as the bubble cursor technique (Grossman & Balakrishnan, 2005).

#### Object Hook

When the pointer moves towards a set of objects, the closest object ahead of the pointer along its movement direction will be “hooked” as the target of interest: a proxy of the object is fixed at the object’s position and the object itself remains moving (Figure 5(d)&e). The user needs to cross the proxy to select the original object (Figure 5(f)). This could form an undemanding selection as the proxy is stationary and also just ahead of the pointer along its movement trajectory.

## Limitation and Future Work

As previous studies of crossing-based selection with stationary targets (Accot & Zhai, 2002; Forlines & Balakrishnan, 2008; Luo & Vogel, 2014; Tu et al., 2019; Wobbrock & Gajos, 2008), our study used circular targets for pointing tasks and bar targets for crossing tasks. While the bar shape is a commonly used target shape for crossing studies, it is meaningful to evaluate user performance of crossing targets with other shapes like circles. This could generalize our results to a greater extent. In addition, we would like to investigate the performance of *crossing* in moving target selection with other input devices, for example, direct finger touch (Luo & Vogel, 2014), virtual reality interaction (Tu et al., 2019), and track-ball input (Yau et al., 2011). It is also of interest to explore crossing performance of selecting a random moving target with the presence of distractors, because such a scenario can be found in realistic applications such as video games and air traffic control applications (Hasan et al., 2011).

## Conclusion

Moving target selection is a common task for many interactive systems. We conducted a fundamental study to look into how users perform target selection with crossing compared to pointing. Results show that crossing generally had advantages over pointing in terms of selection time, accuracy and subjective feedback, with a 12.37% reduction in task completion time and a 5.88% increase in accuracy rate for *orthogonal crossing*, and a similar task time and a 4.71% increase in accuracy rate for *collinear crossing*. Different crossing types (*collinear crossing* vs. *orthogonal crossing*) varied in user performance. Pointing and crossing would have comparable performance when targets become larger than approximately 14.69 mm or move slower than 14.69 mm/s. $T=a+blog_{2}\left(A+\frac{V}{k}\right)-clog_{2}\left(\frac{W}{2}-\frac{V}{k}\right)$ in (Hoffmann, 1991) can be used to model time performance of crossing-based moving target selection. The results provide theoretical support of applying crossing to moving target acquisition.

## Key Points

• We conducted a comprehensive fundamental study to explore crossing performance in moving target selection.
• We analyzed the pros and cons of crossing in the context of moving target acquisition based on experimental data collected.
• Our work contributes new controlled empirical evidence and theoretical results that provide fundamental support for crossing-based moving target selection.
